# Destroying a Treasure

**Published:** 1884-04

**Authors:** 


### DESTROYING A TREASURE.

IN THE *North American Review* Dr. Felix L. Oswald traces the effect upon the climate and physical condition of many lands produced by the destruction of forests. Asia Minor, he says, has become "the epitome of a dying continent." In Spain the agricultural value of the lowlands, which once attracted the Visigoths from their Danubian homes, has been reduced by more than 80 per cent. The coast lands of the Mediterranean have wasted away in a decline "which seems to be the ultimate fate of all civilized countries." "Planets die by dessication, and there is no doubt that the hectic growth of that malady begins to be felt in the lands of the Western Hemisphere." In two centuries the lumbermen of the United States have "killed as many trees as the inhabitants of Southern Europe felled in two thousand years between the foundation of Rome and and conquest of Granada." The water-courses of New England have shrunk until mills which once had ample water-power are compelled to resort to steam. The desiccation which has made barren the vegas of Southern Spain, has set in along our Gulf States.

In fifty years, says Dr. Oswald, if the process continues, our cotton States will be reduced to the necessity of raising their crops by the aid of irrigation. The locust will ravage the plains of the Gulf coast. Agriculture will invade the high valleys of the Alleghanies. The soil of the mountain slopes, stripped of their forests, will be washed away by winter rains and thawing snows. Rivers will shrink to brooks in summer, but flood their valleys in spring; Louisiana and Southern Arkansas will be innundated as regularly as Egypt, and at the mouth of the Mississippi the deposits of river sediment will keep a host of dredge-boats busy. When the stress begins to be felt the people will have to resort to tree-planting to save themselves from starvation.

The writer, leaving the subject of the genesis of deserts, proceeds to consider the history of great floods, and attributes them everywhere to the destruction of forests.

---

### EXPERIMENTS WITH MINERAL WATER UPON DOGS.

FROM experiments upon dogs, Lewaschew and Klikowitch have concluded that the effect of ordinary mineral waters is to increase the quantity of bile and make it more fluid and watery. This increased flow is beneficial in freeing the gall bladder from stagnant bile. The action of artificial solutions of alkaline salts, as well as of hot water, was found to be similar to that of the natural mineral waters.

